# Shifts in Fungal Communities and Potential Functions Under Masson Pine Forest-to-Tea Plantation Conversion in Subtropical China

**DOI:** 10.3390/microorganisms13071614

**Published:** 2025-07-09

**Authors:** Xiaofang Ma, Xiaofang Ou, Dan Chen, Yong Li, Cameron McMillan, Tida Ge, Ji Liu, Min Xue, Cong Wang, Weijun Shen

**Affiliations:** 1 State Key Laboratory for Conservation and Utilization of Subtropical Agro-Bioresources, Guangxi Key Laboratory of Forest Ecology and Conservation, College of Forestry, Guangxi University, Nanning 530004, China; max_0205@163.com (X.M.); 2213200123@st.gxu.edu.cn (X.O.); 20200100@gxu.edu.cn (W.S.); 2 State Key Laboratory for Quality and Safety of Agro-Products, International Science and Technology Cooperation Base for the Regulation of Soil Biological Functions and One Health of Zhejiang Province, Ningbo University, Ningbo 315211, China; getida@nbu.edu.cn; 3 Key Laboratory of Environment Change and Resources Use in Beibu Gulf, Ministry of Education, and Guangxi Key Laboratory of Earth Surface Processes and Intelligent Simulation, Nanning Normal University, Nanning 530001, China; chend2022@163.com; 4 Department of Land, Air and Water Resources, University of California, Davis, CA 95616, USA; cmcmillan@ucdavis.edu; 5 State Key Laboratory of Atmospheric Boundary Layer Physics and Atmospheric Chemistry, Institute of Atmosphere Physics, Chinese Academy of Sciences, Beijing 100029, China; yli@isa.ac.cn; 6 State Key Laboratory of Loess and Quaternary Geology, Institute of Earth Environment, Chinese Academy of Sciences, Xi’an 710061, China; liuji17@mails.ucas.ac.cn

**Keywords:** land-use conversion, fungi, functional category, *Camellia sinensis*, *Pinus massoniana*

## Abstract

The land-use conversion of Masson pine forests to tea fields is extensively practiced across subtropical China, primarily driven by its economic benefit. However, the effects of this conversion on soil fungal communities and functional guilds are poorly understood. Herein, a field experiment was conducted in a Masson pine forest (F), a 5-year-old tea plantation without (FT-CK) fertilization or with (FT-N), and a 30-year-old tea plantation (FT-O) to assess the impact of Masson pine forest-to-tea conversion on soil fungal abundance, community structure, and functional guilds by using qPCR and high-throughput sequencing. Compared to F, fungal abundance significantly decreased by 95%, 68%, and 79% in FT-CK, FT-N, and FT-O, respectively, probably caused by the decreased total nitrogen content and habitat disruption. Fungal alpha diversity significantly increased in FT-N and FT-O compared to FT-CK. FT-O presented the highest percentages of *Mortierella* among treatments, which favours soil organic carbon accumulation. FUNGuild-based predictions showed that FT-CK and FT-N had higher relative abundances of plant pathogens than F and FT-O. FT-O presented the highest percentages of litter and soil saprotrophs but exhibited the lowest percentages of ectomycorrhizal fungi among treatments, likely driven by increased soil organic carbon, total nitrogen, and total phosphorus content. Our findings demonstrate that Masson pine forest-to-tea conversion significantly degrades soil fungal community and function, highlighting the urgent need for soil management strategies (e.g., organic amendments) to enhance soil health in tea agroecosystems.

## 1. Introduction

In 2023, tea (*Camellia sinensis* L.) plantations in China covered 3.43 million ha and produced 3.54 million tons, representing an increase of 1.09% and 5.95% from the previous year [1]. Driven by rising tea demand, converting Masson pine (*Pinus massoniana*) forests to tea fields has become a common land-use change in China [2]. The expansion of tea plantations has raised concerns regarding the long-term ecological consequences on soil health, microbial communities, and carbon and nitrogen cycling [3]. Land-use change alters soil composition and properties (such as pH, soil organic carbon, SOC, and nutrient content), which can be primarily attributed to differences in soil management strategies, distinct aboveground vegetation, and variations in carbon inputs and quality through leaf litter and root exudation [4,5,6]. Converting a Masson pine forest to a tea plantation is likely to accelerate SOC mineralization, potentially diminishing SOC and elevating the soil ammonium-N (NH_4_^+^−N) and nitrate-N (NO_3_^−^−N) contents, alongside inducing alterations in pH [7,8]. The initial stage of converting Masson pine forests to tea plantations often increases soil pH because of elevated basic cations from the decomposing pine biomass [9,10]. Therefore, elucidating the changes in soil fertility and health associated with the conversion of Masson pine forests to tea plantations is essential for understanding the ecological consequences of land-use change, informing sustainable management practices, and promoting the long-term sustainability of tea agroecosystems.

Land-use conversion disrupts not only the physicochemical properties of the soil but also its microbial community composition, including fungi, which play critical roles in soil nutrient cycling, organic matter decomposition, plant growth, and disease suppression [11,12]. Soil fungi occupy diverse and complex niches, use varied ontogenic strategies, and form functional guilds that play vital roles in nutrient cycling [13]. They can be broadly categorized into three major functional guilds: symbiotic, saprotrophic, and pathogenic fungi [14]. Mycorrhizal fungi form symbiotic associations with plant roots, enhance nutrient acquisition, and support plant growth. Saprotrophic fungi decompose organic matter, thereby contributing to nutrient turnover and soil organic carbon dynamics. In contrast, pathogenic fungi negatively affect plant health and community stability [15]. Fungi are of considerable importance in the soil environment for promoting plant growth and supporting ecological stability.

Land-use conversion, such as from rice paddies to upland cultivation or from woodlands to agricultural fields, reshapes fungal community structures by reducing fungal diversity [16,17]. Converting forests to tea plantations enhances the gross rate of nitrification, N mineralization, and NH_4_^+^ immobilization [18], whereas fertilization in tea fields reduces soil fungal diversity and shifts community structure due to alterations in soil characteristics, including pH, NO_3_^−^−N, and total N (TN) [19]. Deforestation of tropical forests for rubber monoculture cultivation reduces SOC and fungal abundance, leading to a shift in fungal communities from r-strategists to K-strategists, resulting in more pathogenic fungi at the expense of saprotrophic and arbuscular mycorrhizal fungi [20]. Palta et al. [21] reported that land-use conversion from forests to either farmland or rangeland led to substantial declines in soil fungal diversity, emphasizing the negative impacts of intensive soil disturbance through tillage and vegetation changes. Although the effects of land use change on soil fungal communities have been widely investigated, previous studies have focused on temperate or tropical broadleaf forest conversions and typical agricultural transitions. However, the effects of converting Masson’s pine forests to tea plantations on soil fungal communities and functional guilds remain poorly understood.

For the theoretical reasons above, two hypotheses were proposed:

(1) The conversion of Masson pine forests to tea plantations would initially increase soil pH but decrease SOC and TN, resulting in a reduction in fungal abundance due to a reduction in litter production and alterations in litter quality.
(2) Fertilization and increasing stand age would increase soil nutrients, potentially changing fungal abundance and community composition and improving fungal functional characteristics (such as inhibition of pathogens and enrichment of beneficial fungi).

To test these hypotheses, a field experiment was conducted to assess the impact of the conversion of Masson pine forests to tea plantations on the soil properties, abundance, community composition, and functional categories of fungi. This study will inform targeted management strategies for tea plantations converted from Masson pine forests to reconcile agricultural productivity with long-term ecological resilience in tea agroecosystems.

## 2. Materials and Methods

### 2.1. Research Site

The study area (Jinjing catchment) is situated within the Changsha Research Station for Agricultural and Environmental Monitoring of the Chinese Academy of Sciences (CAS), Changsha county, Hunan province, China (28°32′50″ N, 113°19′58″ E). This region features a subtropical monsoon climate, with an average annual temperature of 17.5 °C and precipitation of 1389 mm (automatically recorded by a weather station approximately 50 m away from the experimental site). Rainfall is mostly concentrated in April to June and accounts for 47.6% of annual rainfall. The soil was classified as Haplic Alfisol, which developed from a granite parent material. The physicochemical properties of the surface soil (0–20 cm depth) are shown in Figure S1 and Table S1.

### 2.2. Experimental Design

This prolonged field study was initiated in December 2012. The Masson pine forests were approximately 30 years old, with a 40° slope and a mean diameter at breast height (DBH) of 31.28 cm, all with the same soil type. The plantation had tree canopy coverage of 92% and a leaf area index (LAI) of 2.24. The experiment consisted of four treatments with three replicates each. Briefly, nine Masson pine forest plots, each occupying an area of 30 m in length and 20 m in width, were initially selected, of which six were converted to tea fields through a series of forest clearances and terrace constructions in January 2013, leaving the remaining three plots as Masson pine forest (F). Following conversion, the newly established tea fields were randomly assigned to two treatments with three replicates each: conventional fertilizer treatment (FT-N) and non-fertilization treatment (FT-CK) (Figure S2). Additionally, three plots in a 30-year-old tea field (FT-O) converted from a Masson pine forest with identical parent soil material were established. Contour planting was applied in the tea field, and the standard rate of nitrogen fertilizer application was 450 kg N ha^−1^ yr^−1^, which consisted of 67% urea added from March to May and 33% oilseed residue added from October to December. Further details regarding fertilization amounts and planting schedules can be found in [10].

### 2.3. Soil Sampling and Physicochemical Properties Analysis

Soil was sampled in early December 2017 (the non-growing season for tea trees). Following an S-shaped sampling pattern, ten cores from each plot were collected from the 0–20 cm layer with a diameter of 3 cm. The samples were mixed and homogenized to form composites for analysis. Each sample was evenly partitioned in triplicate and passed through a 2.0 mm sieve after removing discernible gravel and vegetative debris. Each composite soil sample was then divided into three subsamples: (1) a refrigerated subsample stored at 4 °C for the analysis of biological and abiotic properties, including NH_4_^+^−N, NO_3_^−^−N, microbial biomass carbon (MBC), nitrogen (MBN), and soil heterotrophic respiration (Rh); (2) an air-dried subsample for physicochemical characterization of soil pH, SOC, TN, and total phosphorus (TP); and (3) a cryopreserved subsample (approximately 200 g) flash-frozen in liquid nitrogen and preserved at −80 °C for DNA extraction. Soil respiration (Rh) was measured by the static chamber method [10]. Gas samples were determined using an Agilent 7890A gas chromatograph (Agilent Technologies, Palo Alto, CA, USA). The comprehensive details regarding the measurement of different soil physicochemical characteristics, such as soil pH, soil bulk density (BD), SOC, TN, TP, NH_4_^+^−N, NO_3_^−^−N, MBC, and MBN, are outlined in the Supplementary Materials [22,23,24,25].

### 2.4. Soil Microbial Analysis

#### 2.4.1. Soil DNA Extraction and Illumina Sequencing

Total DNA was extracted from fresh soil samples (0.5 g) using a PowerSoil DNA Isolation kit (MoBio Laboratories, Carlsbad, CA, USA), following the procedure suggested by the manufacturer’s instructions. DNA concentration and purity were assessed using 1% agarose gel electrophoresis, followed by quantification using a NanoDrop ND-1000 spectrophotometer (NanoDrop Technologies, Wilmington, DE, USA). All DNA sample concentrations exceeded the 5 ng μL^−1^ threshold, making them suitable for downstream sequencing and quantitative polymerase chain reaction (qPCR). Sequencing of the purified amplicons was performed on an Illumina MiSeq platform (Illumina Inc., San Diego, CA, USA) at Majorbio Bio-Pharm Technology Co., Ltd. (Shanghai, China) in the paired-end mode (2 × 250 bp for fungal internal transcribed spacer amplicons). All the raw sequences were uploaded to the NCBI database (accession number: PRJNA1270435).

#### 2.4.2. Quantitative Polymerase Chain Reaction

The 18S rRNA gene abundance was measured using quantitative polymerase chain reaction (qPCR), utilizing the primer set NS1 (5′-ATTCCCCGTTACCCGTTG-3′)/Fung (5′-GTAGTCATATGCTTGTCTC-3′) on an Applied Biosystems thermocycler (Beverly, MA, USA) [26]. The PCR amplification was executed in a 10 μL reaction volume that contained 1 μL template DNA (5–10 ng), 5 μL of SYBR Green PCR Master Mix (TaKaRa Bio Inc., Shiga, Japan), 0.2 μL Rox Reference Dye II, 0.3 μL of each of the forward and reverse primers (10 μM), and 3.2 μL sterilized deionized water. Triplicate reactions were performed in the qPCR analysis. Standard curves were generated by plotting the log value of plasmid DNA (10× stepwise dilutions of DNA containing 18S genes) against the Ct number. The reaction efficiencies were 97–109%, and R^2^ was 0.996–0.999.

#### 2.4.3. Sequence Analysis

Quality trimming, merging, and assignment of raw sequencing reads were performed using QIIME2 [27]. Amplicon sequence variants (ASVs) were filtered to ensure data quality, excluding those with confidence values below 0.8 (indicating low sequence reliability) and read counts of less than 10 (indicating low abundance or potential sequencing errors). Taxonomic annotation of fungal sequences was conducted using the UNITE database (https://unite.ut.ee/; accessed on 21 July 2024. Functional roles of the fungal community, such as saprotrophy, pathogenicity, and symbiosis, were assigned based on nutrient acquisition strategies using the FUNGuild algorithm [28]. Functional groups were categorized into guilds according to varying degrees of confidence in “highly probable”, “probable”, and “possible”.

### 2.5. Statistical Analysis

All statistical analyses were performed using R (version 4.3.1). An analysis of variance (*p* < 0.05) was conducted to assess significant differences in environmental factors, fungal abundance, and diversity. The similarity of fungal communities across treatments was assessed using Bray–Curtis-based principal coordinate analysis (PCoA) with the ‘vegan’ package in R. The environmental fit (envfit) function provided by the vegan package in R was applied to correlate PCoA axes with the relative abundances of each dominant fungal genera. Vectors of dominant fungal genes were fitted to the ordination data with vegan’s envfit function with 999 permutations; only the factors with *p* < 0.05 were retained and plotted as vectors, and their lengths represented the explanatory intensity (R^2^). For the fungal community composition and frequency of fungal functional groups that were identified as non-normally distributed, a non-parametric approach was used. The Kruskal–Wallis test was conducted, followed by Dunn’s post-hoc test for multiple comparisons to identify significant inter-treatment differences. To elucidate the relationships among fungal abundance, dominant soil fungal genera, fungal functional groups, and soil characteristics—including Rh rate, pH, BD, SOC, TN, TP, NH_4_^+^−N, NO_3_^−^−N, MBC, and MBN contents—within Masson pine forests and tea plantations, Pearson’s correlation analysis and transformation-based redundancy analysis (tb-RDA) were used. For the relationships between the functional groups and soil properties, tb-RDA was used in the R package, with the relationships examined with Monte Carlo permutations (999 repetitions). The relative importance of environmental variables in driving fungal abundance and diversity was evaluated using random forest analysis, as implemented in the R package ‘randomforest’. The significance of each variable was quantified by the percentage increase in mean squared error (% IncMSE), as derived from the ‘importance’ function within the analysis. Associations of fungal dominant phyla with soil environment factors were evaluated by the Mantel test using the ‘mantel’ function. In this study, the statistical significance and correlation thresholds were set at *p* < 0.05.

## 3. Results

### 3.1. Soil Environmental Factors

The conversion from Masson pine forests to tea fields resulted in substantial alterations in soil environmental factors (Figure S1). Compared to Masson pine forests (F), soil pH increased in the non-fertilization treatment (FT-CK) and FT-N treatments (FT-N) but decreased in the 30-year-old tea fields (FT-O) (*p* < 0.05). Soil pH in FT-N and FT-O decreased significantly compared to that in FT-CK (*p* < 0.05). The BD in FT-O was significantly lower than that in FT-CK, indicating that the soil BD of tea plantations that changed from forests decreased with planting age. SOC and TN contents under the FT-N treatment were lower than those under the F and FT-O treatments and greater than those under FT-CK (*p* < 0.05). The TP concentrations in the F, FT-CK, and FT-N treatments decreased by 56.6%, 68.3%, and 53.3%, respectively, compared to FT-O. MBC was significantly higher in FT-CK than in the F, FT-N, and FT-O treatments (*p* < 0.05). FT-N and FT-O contained higher concentrations of MBN, NH_4_^+^−N, and NO_3_^−^−N than F and FT-CK. The F, FT-N, and FT-O treatments increased Rh markedly by 2.55-, 4.46, and 3.88 times relative to FT-CK.

### 3.2. Soil Fungal Abundance

**Figure 1:**
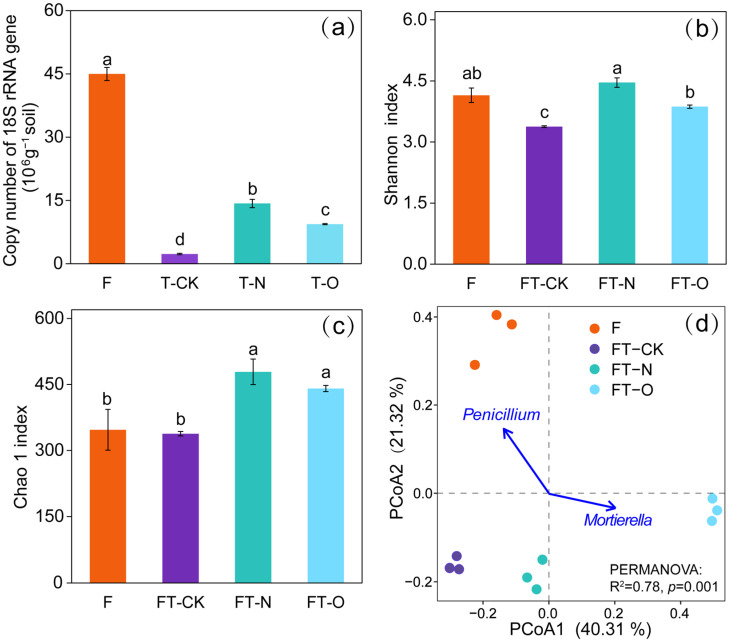
Abundance of 18S rRNA genes: (**a**) Shannon index, (**b**) Chao 1 index, and (**c**) principal coordinates analysis (PCoA) (**d**) of soil fungal communities in F, FT-CK, FT-N, and FT-O treatments. Significant differences are indicated by lowercase letters according to Duncan’s test (*p* < 0.05). The vertical bars indicate standard errors (*n* = 3). PCoA was based on Bray–Curtis similarities of fungi community compositions. Individual lines with an arrow indicate highly correlated fungal genes (Envfit analysis in R package, vegan: R^2^ = 0.78, *p* = 0.001) with ordination Masson pine forest (F; 30-year-old); 5-year-old tea plantation with fertilization (FT-N) or without (FT-CK) fertilization; 30-year-old tea plantation (FT-O).

Fungal abundance varied significantly across all treatments, with the highest value observed in F (44.98 × 10^6^ ± 1.55 × 10^6^ copies g^−1^ soil), followed by FT-N (14.29 × 10^6^ ± 0.99 × 10^6^ copies g^−1^ soil), FT-O (9.38 × 10^6^ ± 0.13 × 10^6^ copies g^−1^ soil), and FT-CK (2.32 × 10^6^ ± 0.17 × 10^6^ copies g^−1^ soil) (Figure 1a). Compared to F, fungal abundance in the FT-CK, FT-N, and FT-O treatments decreased by 94.85%, 68.23%, and 79.14%, respectively. In contrast, compared with FT-CK, fungal abundance increased by 516.42% in FT-N and 304.7% in FT-O (Figure 1a) (*p* < 0.05).

### 3.3. Soil Fungal Diversity and Community Composition

#### 3.3.1. Soil Fungal Diversity

The alpha diversity of the soil fungal communities differed between treatments (Figure 1b,c). Compared to FT-CK, the Shannon index in F, FT-N, and FT-O increased by 22.80%, 32.05%, and 14.55%, respectively, and FT-N and FT-O treatments increased the Chao 1 index by 41.63% and 30.40%, respectively (*p* < 0.05).

PCoA ordination showed that soil fungal community structures differed significantly among treatments, with the first and second axes accounting for approximately 40.31% and 21.32% of the total variation in fungal community composition, respectively (Figure 1d) (*p* = 0.001). Correlation analysis between the first two axes and the relative abundance of the dominant fungal genera further suggested that the shift in fungal community structure was mainly associated with changes in the relative abundance of *Penicillium* and *Mortierella*.

#### 3.3.2. Soil Fungal Community Composition

**Figure 2:**
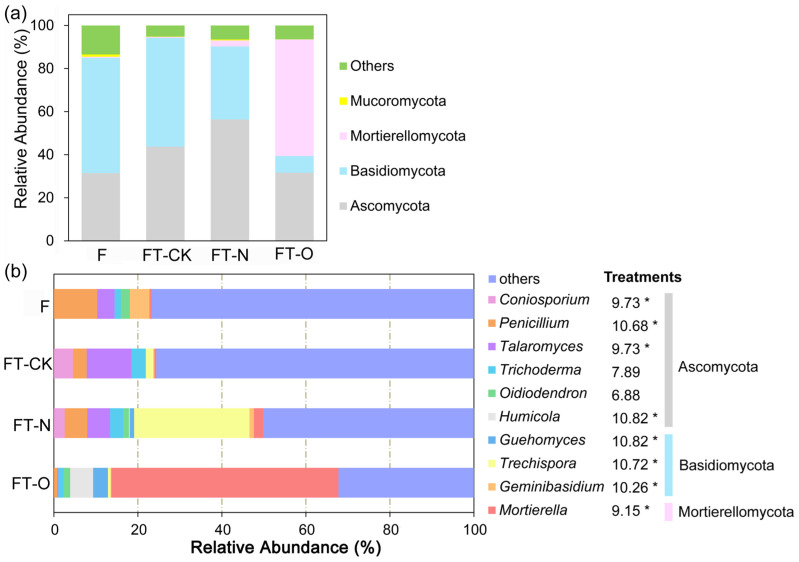
Soil fungal community composition relative abundance (%) of the four most abundant fungal phyla (**a**) and relative abundance of the top 10 genera (**b**) in different treatments. Kruskal–Wallis tests showed the effect of treatments on the relative genera of fungi (* *p* < 0.05).

Taxonomic classification at the phylum level indicated the presence of high levels of Mucoromycota, Mortierellomycota, Basidiomycota, and Ascomycota in all soil samples (Figure 2a). The fungal communities in the F, FT-CK, and FT-N treatments were predominantly composed of Ascomycota and Basidiomycota, with combined relative abundances of 84.94–94.27% across these treatments. However, under the FT-O treatment, Mortierellomycota and Ascomycota were the dominant phyla, accounting for 85.77% of the total abundance.

The composition of the fungal community varied between treatments at the genus level (Kruskal–Wallis, *p* < 0.05; Figure 2b). The 10 dominant genera were *Coniosporium*, *Penicillium*, *Talaromyces*, *Trichoderma*, *Oidiodendron*, *Humicola*, *Guehomyces*, *Trechispora*, *Geminibasidium,* and *Mortierella*. The most dominant genus in the F treatment was *Penicillium* (10.24%)*,* followed by *Geminibasidium* (4.65%)*. Talaromyces* (10.65%) and *Trechispora* (27.45%) were the most dominant genera in the FT-CK and FT-N treatments, respectively. The most dominant genus in the FT-O treatment was *Mortierella*, accounting for 54.09%. Compared to FT-CK, *Penicillium* and *Geminibasidium* were reduced by 96.30% and 92.36%, respectively, in FT-O (*p* < 0.05). Conversely, *Mortierella* was 128.51-fold greater in FT-O than in FT-CK.

### 3.4. Soil Fungal Functional Profiles

**Figure 3:**
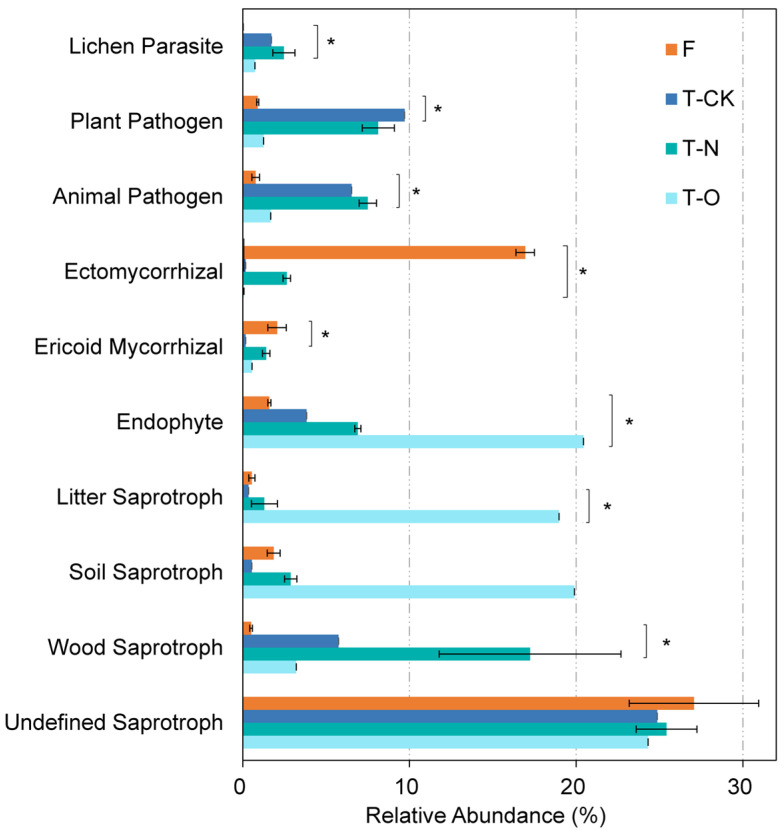
Relative abundance (%) of the 10 most dominant fungal functional groups under the four treatments. The Wilcoxon rank-sum test was carried out to examine differences in the relative abundance of the dominant fungal functional groups within different treatments according to *p* < 0.05 after Bonferroni correction (* *p* < 0.05).

Utilizing the FUNGuild database for taxonomic classification, the effect of land-use change from Masson pine forests to tea fields on the relative abundance of diverse functional guilds was evaluated. The top 10 fungal functional groups, which included lichen parasites, plant pathogens, animal pathogens, ectomycorrhizal fungi, ericoid mycorrhizal fungi, endophytes, litter saprotrophs, soil saprotrophs, wood saprotrophs, and undefined saprotrophic fungi, were identified. The key functional groups varied across treatments (Kruskal–Wallis, *p* < 0.05; Figure 3). Relative to F, FT-N led to a significant increase in the relative abundance of lichen parasites (179.33-fold) and animal pathogens (8.68-fold), respectively, as well as a substantial enhancement in wood saprotrophs (33.53-fold) (Kruskal–Wallis, *p* < 0.05). Compared to FT-CK, F reduced the proportion of plant pathogens by 90.78% and substantially increased the abundance of ericoid mycorrhizal fungi by 1087.48%. Additionally, endophytes decreased markedly in FT-O compared to F, whereas ectomycorrhizal fungi increased.

### 3.5. Relationships Among Fungal Abundance, Community Composition, Functional Groups, and Soil Characteristics

**Figure 4:**
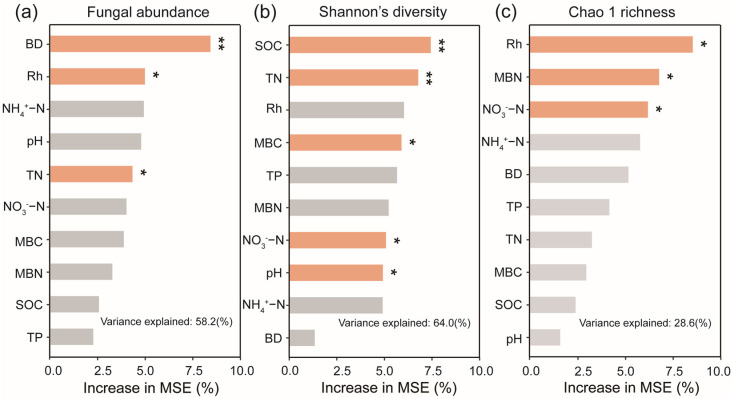
Predictive importance of each soil environmental factor to (**a**) fungal abundance, (**b**) Shannon’s diversity, (**c**) Chao 1 richness, determined using a random forest model (RF). RF importance = increase in % mean square error. Colored and grey columns represent *p* < 0.05 and *p* > 0.05, respectively; * *p* < 0.05, ** *p* < 0.01.

**Figure 5:**
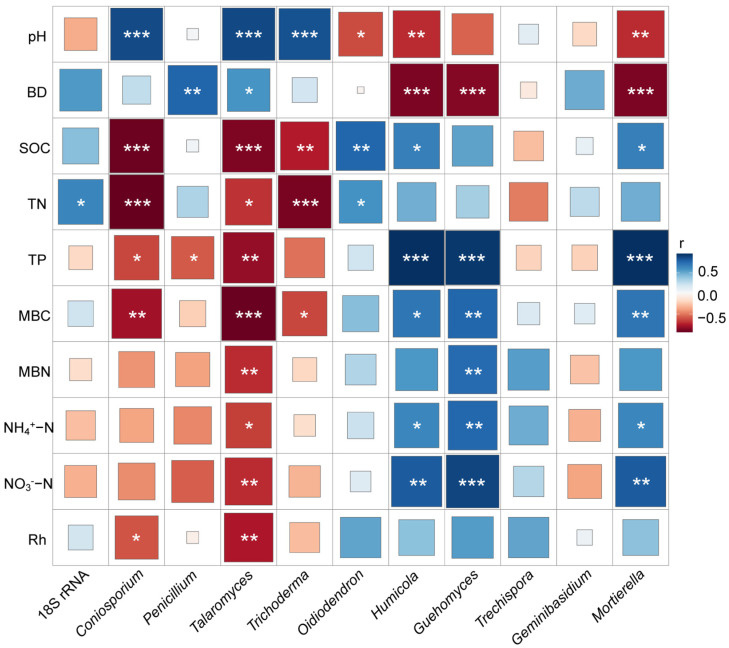
Matrix of Pearson’s correlation analysis of soil environmental factors and fungal communities after land-use conversion from Masson pine forest to tea fields. * *p* < 0.05, ** *p* < 0.01, *** *p* < 0.001.

Environmental variables accounted for 58.2% of the total variance (random forest; Figure 4a). The most important factors affecting soil fungal abundance were BD (8.40%), Rh (4.96%), and TN (4.31%). Pearson’s correlation analysis indicated a positive correlation between soil fungal abundance and TN content (*p* < 0.05) (Figure 5).

The random forest analyses identified SOC (7.44%), soil TN (6.78%), MBC (5.90%), NO_3_^−^−N (5.08%), and pH (4.92%) as the primary predictors of soil fungal Shannon diversity (Figure 4b). The Chao 1 richness was mainly dependent on Rh (8.57%), MBN (6.81%), and NO_3_^−^−N (6.22%) (Figure 4c).

The Mantel test indicated that pH, SOC, and TN were the determinants of Ascomycota abundance among the treatments (Figure S3). The Basidiomycota were regulated by pH, BD, SOC, TP, MBC, MBN, NH_4_^+^−N, and NO_3_^−^−N. Mantel tests demonstrated that Mortierellomycota were highly correlated with BD, TP, MBN, NH_4_^+^−N, and NO_3_^−^−N (*p* < 0.001), followed by pH, SOC, and Rh. Mucoromycota were regulated by pH, BD, SOC, TP, MBC, and NO_3_^−^−N.

To identify the key environmental drivers of fungal community composition, Pearson’s correlation analysis was used to assess the relationships between dominant fungal genera and various environmental parameters (Figure 5). The relative abundance of *Coniosporium* demonstrated a strong positive correlation with pH and a negative correlation with SOC, TN, TP, MBC, and Rh. There was a strong positive correlation between *Penicillium* and BD, and a negative correlation between *Penicillium* and TP. *Talaromyces* had a strong positive correlation with pH and BD, and a negative correlation with SOC, TN, TP, MBC, MBN, NH_4_^+^−N, NO_3_^−^−N, and Rh. A strong positive correlation was observed between *Trichoderma* and pH, whereas negative correlations were observed with SOC, TN, and MBC. *Oidiodendron* abundance was negatively correlated with soil pH and positively correlated with TN and SOC. Both *Humicola* and *Mortierella* had strong negative correlations with pH and BD and were positively correlated with SOC, TP, MBC, NH_4_^+^−N, and NO_3_^−^−N. *Guehomyces* displayed a negative correlation with BD and positive correlations with TP, MBC, MBN, NH_4_^+^−N, and NO_3_^−^−N.

**Figure 6:**
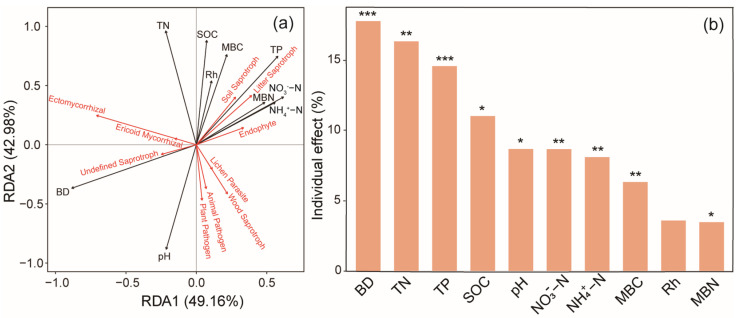
Transformation-based redundancy analysis (tb-RDA) estimated the relationships among soil environmental factors and the relative abundance of the (**a**) dominant fungal functional groups. (**b**) Bar plot of the individual effects of soil properties in fungal functional groups. * *p* < 0.05, ** *p* < 0.01, *** *p* < 0.001.

The tb-RDA explained 97.96% of the total variation in the dominant fungal functional groups, with the first two axes explaining 88.49% of this variation (Figure 6a). In addition, BD, TN, SOC, TP, pH, NO_3_^−^–N, NH_4_^+^−N, MBC, and Rh were strongly correlated with the dominant fungal functional groups, contributing 19.12%, 16.44%, 13.75%, 9.12%, 9.01%, 7.75%, 7.22%, 7.00%, and 5.96% of the explained variation, respectively (Figure 6b). pH was negatively correlated with the percentages of endophytes, litter saprotrophs, and soil saprotrophs but was positively correlated with those of plant pathogens, animal pathogens, wood saprotrophs, and lichen parasites. TN, SOC, and TP were negatively correlated with the percentages of plant pathogens and animal pathogens but were positively correlated with those of litter saprotrophs and soil saprotrophs.

## 4. Discussion

### 4.1. Conversion from Masson Pine Forest to Tea Field Decreased Soil Fungal Abundance

The conversion of Masson pine forest to tea plantations substantially reduced soil fungal abundance, driven by three interconnected mechanisms. First, deep plowing before establishing the tea plantations mixed the surface and subsoil layers, which redistributed fungal communities from organic-rich topsoil to nutrient-poor deeper horizons [29,30]. Secondly, the agricultural transition (Masson pine forest converted to tea fields) fragmented fungal habitats, resulting in diminished fungal dispersal and niche availability, which induced a considerable reduction in fungal abundance [31]. Additionally, converting Masson pine forest to tea fields reduced aboveground litter through the removal of felled trees [10], which diminished fungal abundance, as litter is a vital substrate for fungal growth [32]. Conventional fertilization (FT-N) partially mitigated these effects, increasing fungal abundance by 516% compared to FT-CK (Figure 1a). This recovery likely stems from the dual roles of oilseed residues, which act as both nutrient sources (through nitrogen release) and labile organic substrates that stimulate fungal growth [33]. These findings imply that applying N fertilizer (e.g., oilseed residues) during the early stages of tea plantation establishment could mitigate the loss of soil fungal abundance triggered by land-use conversion. As a low-cost and ecologically-compatible practice, this offers a viable strategy for maintaining soil ecological functions in newly converted tea fields, balancing agricultural productivity with fungal conservation.

### 4.2. Conversion from Masson Pine Forest to Tea Field-Induced Changes in Fungal Communities

In our study, the FT-CK treatment resulted in a reduction in the Shannon index of soil fungi compared with the F treatment (*p* < 0.05) (Figure 1b), primarily because of the soil structural disruption and nitrogen leaching induced by deep plowing. The land-use conversion typically destroyed the soil aggregate structure via physical disturbances, in favor of water infiltration and nitrogen leaching [34], ultimately depleting NH_4_^+^−N and NO_3_^−^−N availability (Figure S1h,i), which are the key nutrients governing fungal proliferation and diversity [35]. Pearson’s correlation analysis validated the results, indicating a positive relationship between the Chao1 index and the concentrations of NH_4_^+^−N and NO_3_^−^−N (Figure S4). Intensive management practices (e.g., weeding and irrigation) in tea plantations further reduce plant diversity and carbon inputs, exacerbating resource constraints, thereby diminishing carbon pools accessible to soil fungi and ultimately limiting fungal diversity potential [5,32,36]. Conversely, conventional fertilization (FT-N) alleviated nitrogen limitation, with Shannon and Chao1 indices increasing by 32.05% and 41.63%, respectively, compared to FT-CK. This recovery was driven by oilseed residue inputs, which enriched labile carbon and nitrogen substrates, thereby enhancing fungal diversity [37], as confirmed by the random forest analysis (Figure 4b). However, long-term tea cultivation (FT-O) reversed these gains, with Shannon diversity declining by 13.25% compared to FT-N (*p* < 0.05; Figure 1b). This decline was driven by progressive soil acidification and Al^3+^ accumulation associated with the increasing age of the tea plantation, which suppressed acid-sensitive fungal taxa and ultimately reduced community diversity [29,38].

The dominant fungal phyla exhibited distinct responses to land-use conversion. Basidiomycota, K-strategists specializing in decomposing recalcitrant organic matter, declined by 33.01% in FT-N compared to FT-CK (Figure 2a), likely due to the fertilization of tea gardens, which reduced the soil pH and inhibited the growth of Basidiomycota [39]. Ascomycota, which have been roughly categorized as r-strategists and accepted as saprotrophs [40], were the most predominant fungal phylum in FT-N among the four treatments (Figure 2a). This is likely because fertilization (especially oilseed residues) promotes the proliferation of saprotrophic fungi and enhances their dominance through rapid resource acquisition, ultimately causing a decrease in the abundance of Basidiomycota, which are primarily non-saprotrophic taxa [41]. This finding is in line with previous research indicating that Ascomycota dominance is enhanced in N-fertilized soils [42]. Our findings were confirmed by Mantel tests, in which the percentage of Ascomycota was highly correlated with TN content (Figure S3). Soil organic matter (SOM) quality, particularly its labile carbon fraction, has emerged as a pivotal regulator of fungal community composition. Our results indicated that fertilizer addition elevated Rh by 4.46-fold compared to unfertilized fields (Figure S1h), potentially indicating accelerated microbial utilization of labile C from oilseed residues. This aligns with the dominance of r-strategist fungi in FT-N soils (Figure 2a), which preferentially decompose labile substrates to facilitate rapid growth [43]. There was a reduction in the relative abundance of Ascomycota and Basidiomycota, whereas Mortierellomycota exhibited an increase under the FT-O treatment in comparison to FT-CK. The shift from a high abundance of Basidiomycota and Ascomycota to Mortierellomycota (saprotrophic taxa) occurred in response to modifications in soil environmental factors driven by increased resource availability, mirroring a change in fungal functional groups with advancing stand age [10]. This was also supported by the correlation between Mortierellomycota and N availability (*p* < 0.05) (Figure S3). Mucoromycota, a phylum distinguished by its plant-associated capabilities and encompassing symbiotic mycorrhizal fungi [44], decreased by 68.2% in FT-CK compared with F (*p* < 0.05) (Figure 2a). This decline likely reflects disrupted plant–fungal mutualism in monocultured tea plantations, where reduced plant diversity and altered root exudates may limit symbiotic interactions.

The conversion of Masson pine forests to tea plantations reshaped the soil fungal communities at the genus level, with taxonomically specific responses reflecting adaptations to contrasting management practices. *Trechispora*, a copiotrophic genus thriving in nitrogen-enriched environments, dominated the FT-N treatment, with a relative abundance of 17.22% (Figure 2b), likely because of the elevated nitrogen availability from urea and oilseed residues, which provide preferential substrates for growth [45,46]. *Penicillium*, a primary saprophyte capable of degrading lignin, polyphenols, and root exudates, plays a critical role in enhancing soil fertility and serves as a bioindicator of soil health in tea plantations [47]. The relative abundance of *Penicillium* increased by 63.72% in the FT-N compared to the FT-CK treatment (*p* < 0.05; Figure 2b), likely due to nitrogen enrichment from urea and oilseed residues, which stimulate microbial activity and phenolic acid degradation, thereby improving soil health [48]. Field trials could optimize *Penicillium*-enriched organic amendments (e.g., composted oilseed residues) to accelerate the recovery of newly converted tea soils. *Mortierella* enhances phosphorus uptake by plants through synergistic interactions with mycorrhizal fungi [49]. In addition, *Mortierella*, a saprophytic genus with a strong ability to decompose plant remnants and exploit phenolic compounds [50], dominated FT-O with a relative abundance of 54.09% (Figure 2b). This dominance was driven by multiple factors: (1) soil acidification and Al^3+^ accumulation, which suppressed acid-sensitive competitors; and (2) increased SOC and phenolic acid concentrations from the aging tea plantation, which provided preferential substrates for *Mortierella* proliferation. These findings were further supported by Pearson’s correlation analysis (Figure 5), highlighting *Mortierella*’s adaptation to long-term tea cultivation, where resource specialization and stress tolerance converge to shape the fungal community assembly. Crucially, the dominance of *Mortierella* (54.09%) in long-term tea fields (Figure 2b), and its negative correlation with pathogen abundance align with known antibiotic production capabilities [51]. This supports the development of *Mortierella*-based biofertilizers to suppress soil-borne diseases (e.g., root rot and stem canker) in tea plantations, potentially reducing chemical pesticide use by 30–50%, as demonstrated in vegetable systems [46,47]. The lower relative abundance of *Mortierella* in FT-CK and FT-N than in FT-O may be attributed to the disruption of hyphal networks during the initial intensive management practices (e.g., cultivation, fertilization, and weeding) associated with land use conversion, which compromise fungal resilience and growth [52]. Nitrogen fertilization in the FT-N treatment likely enhanced microbial activity, accelerating the degradation of phenolic acids and reducing their concentration compared to FT-CK. This aligns with the findings that phenolic acid accumulation increases *Talaromyces* but decreases *Guehomyces* [53], explaining the decreased relative abundance of *Talaromyces* and the concurrent increase in *Guehomyces* observed in FT-N (Figure 2b). According to the oligotrophic–copiotrophic theory, nitrogen enrichment favors copiotrophic taxa (e.g., *Guehomyces*) over oligotrophic taxa (e.g., *Trichoderma*) [45,54]. Similar results were obtained using Pearson’s correlation analysis, which demonstrated a negative correlation between the percentages of *Trichoderma* and NO_3_^−^−N content, whereas *Guehomyces* was positively correlated with both NH_4_^+^−N and NO_3_^−^−N content (*p* < 0.05; Figure 5).

### 4.3. Impacts of Conversion from Masson Pine Forest to Tea Field on Soil Fungal Functional Groups

Land use conversion from Masson pine forests to tea plantations fundamentally restructured fungal functional guilds, with distinct shifts in pathogenic, symbiotic, and saprotrophic groups (Figure 3). An increased relative abundance of plant pathogens in FT-CK and animal pathogens in FT-N was observed after land use conversion (*p* < 0.05; Figure 3). This aligns with the reduced SOC contents as a result of these treatments, as the reduction in organic matter has been shown to weaken pathogen suppression by antagonistic fungi [55]. RDA and Pearson’s correlation analyses further confirmed the negative correlation between pathogen abundance and SOC (Figure 6a and Figure S4). Li et al. [56] reported that *Mortierella* could produce antibiotic compounds that can combat a range of soil-borne diseases. Here, a reduced relative abundance of plant and animal pathogens was found in FT-O compared to FT-CK and FT-N (Figure 3). This can be explained by the substantial increase in *Mortierella,* which leads to the secretion of disease-resistant substances in FT-O, thereby suppressing the growth of pathogenic fungi. F and FT-O substantially decreased plant pathogen abundance across all treatments, which was possibly mediated by the enhanced growth of Masson pine and tea trees. This growth promotion mainly stems from increased SOC and TN (Figure S1c,d), which could further strengthen plant innate disease resistance [57]. The RDA and Pearson’s correlation analyses demonstrated a strong negative correlation between pathogen abundance and SOC and TN concentrations (Figure 6 and Figure S4), suggesting that increasing the SOC and TN concentrations inhibited pathogen growth. Symbiotic endophytic fungi, which reside within plant tissues, improve host growth and enhance tolerance to abiotic and biotic stresses (e.g., those caused by drought and pathogens) by obtaining nutrients from their hosts [58]. The FT-O treatment exhibited the highest endophytic fungal abundance of all the treatments (Figure 3), which may be attributed to the coevolutionary relationship between plants and their endophytic fungal partners [59]. In contrast, treatment F resulted in the lowest endophytic fungal abundance (*p* < 0.05) (Figure 3), likely because the conversion of Masson pine forests to tea fields altered the ecological niche for endophytic microorganisms. Fungal–host interactions are equilibrated and influenced by environmental factors [60]. Mutations in endophytes can alter their symbiotic roles, potentially shifting their ecological lifestyles from mutualistic to pathogenic or vice versa [61]. The F treatment showed 95.79-fold, 5.42-fold, and 244.03-fold higher relative abundance of ectomycorrhizal fungi than the FT-CK, FT-N, and FT-O treatments, respectively (Figure 3), suggesting a trade-off between endophytic and ectomycorrhizal fungal communities under different land use practices. Ectomycorrhizal fungi protect plants against root pathogens by forming a physical barrier that encircles and protects the roots, preventing pathogens from accessing infection sites and inhibiting their access to photosynthates [62]. The relative abundance of litter and soil saprotrophs increased with tea plantation stand age (Figure 3). This potential function shift likely reflects dual pathways of carbon transformation: while the accumulated plant residues and root exudates enhance labile C pools, serving as the primary energy source fueling saprotroph proliferation [28,42], concurrent microbial processing progressively converts these inputs into recalcitrant SOM through necromass accumulation and mineral association [63]. Consequently, in older tea plantations, the co-accumulation of labile C (supporting microbial activity) and recalcitrant SOM (enhancing physical stability) synergistically improves overall SOM quality, thereby promoting both biological functions and long-term carbon sequestration. This was supported by the positive correlations among SOC, soil saprotrophs, and litter saprotrophs (Figure 6a and Figure S4). Saprophytic fungi secrete an array of hydrolytic and oxidative enzymes that aid in the decomposition of dead or senescent plant materials, thereby enhancing soil organic matter [13]. Likewise, fertilizer (urea and oilseed residues) addition increased the SOC content, thus increasing substrates for saprotroph growth, leading to higher percentages of wood saprotrophs and soil saprotrophs in FT-N than in FT-CK (Figure 3 and Figure S2).

### 4.4. The Present Research Applications and Future Research Prospects

In the present study, we investigated soil fungal abundance and community composition in response to land use conversion. Our study underscores two immediate applications: firstly, enhancing biocontrol against fungal pathogens and insect pests in the early stage of forest-to-tea conversion through deployment of fungal biocontrol agents (e.g., *Trichoderma*, *Mortierella*) harnesses its pathogen-suppressive traits to reduce agrochemical dependency. Secondly, SOC content in Chinese tea plantations is typically 10–20 g kg^−1^ [64], yet SOC content (4.5–8.64 g kg^−1^) falls below the nationwide mean during the initial conversion from forest to tea plantation (Figure S1c). Tea production sectors can implement strategic fertilizer supplementation (e.g., oilseed residue application) to mitigate fungal diversity loss during forest-to-tea conversion. However, the long-term application of nitrogen fertilizer leads to soil acidification in tea plantations (Figure S1a). The use of green fertilizers, such as biochar, organic fertilizers, and microbial agents, may serve as effective methods to alleviate soil acidification [65] while simultaneously increasing tea plant productivity and soil nutrient content. This indicates that further research is necessary to quantify the synergistic effects of applying biochar, organic fertilizers, and biofertilizers during forest-to-tea plantation conversion to improve (i.e., help alkalinize) acidified soils, restore fungal functionality, and enhance ecosystem services. A deeper understanding of such knowledge will provide a rationale for improving tea field soil health and promoting the sustainable development of the tea industry.

## 5. Conclusions

The results of our study suggested that the conversion of Masson pine forests to tea plantations restructured the soil fungal communities and functional guilds by changing the soil properties. The initial conversion reduces fungal abundance and alpha diversity, probably due to the loss of TN and habitat disruption. Fertilization effectively reverses this trend by increasing fungal abundance and alpha diversity, mainly due to the increase in SOC and TN content. Land-use conversion promoted plant pathogens, likely due to the decreased SOC content, while long-term tea plantations suppressed pathogens via *Mortierella* enrichment and SOC and TN accumulation. Future research should prioritize experimental investigations of soil improvement managements (e.g., the application of green manure and biochar) under Masson pine forest-to-tea plantation conversion. Promising treatments should be subsequently evaluated for their effects on soil microbial communities and functional dynamics.

## Data Availability

The data that support the findings of this study are available from the corresponding author upon reasonable request.

## Supplementary Materials

The following supporting information can be downloaded at https://www.mdpi.com/article/10.3390/microorganisms13071614/s1, Table S1: The granulometric composition of the soil in different treatments; Figure S1: Soil environmental properties; Figure S2: (a) and (b) Geographical location of the study area in Changsha County, Hunan Province, China; (c) Field experimental plots using a randomized block design with three replicates; (d) Photos of the field experiment in study; Figure S3: Associations among soil environmental factors and abundant fungi phylum in four treatments. Figure S4: Pearson correlations (r) between soil environment factors and soil fungal alpha diversity (Shannon and Chao 1 index), the relative abundance of dominant fungal phyla and functional groups.
