# Supplementary material for: Shifts in Fungal Communities and Potential Functions Under Masson Pine Forest-to-Tea Plantation Conversion in Subtropical China
Source: Microorganisms. 2025 Jul 9;13(7):1614. doi: 10.3390/microorganisms13071614 (PMC12299667; doi:10.3390/microorganisms13071614)
Supplement: Supplementary file 1 [file microorganisms-13-01614-s001.zip › microorganisms-3614209-supplementary.pdf]

Supplement Materials

Title：Shifts in Fungal Communities and Potential Functions under Masson pine Forest-to-tea Plantation Conversion in Subtropical China

Authors：**Xiaofang Ma[1, 5], Xiaofang Ou[1], Dan Chen[2, 4], Yong Li[3], Cameron McMillan[4], Tida Ge[5], Ji Liu[6], Min Xue[2*], Cong Wang[1, 4*], Weijun Shen[1]**

Author Affiliations：

[1] State Key Laboratory for Conservation and Utilization of Subtropical Agro-Bioresources, Guangxi Key Laboratory of Forest Ecology and Conservation, College of Forestry, Guangxi University, Nanning 530004, China

[2] Key Laboratory of Environment Change and Resources Use in Beibu Gulf Ministry of Education and Guangxi Key Laboratory of Earth Surface Processes and Intelligent Simulation, Nanning Normal University, Nanning 530001, China

[3] State Key Laboratory of Atmospheric Boundary Layer Physics and Atmospheric Chemistry, Institute of Atmosphere Physics, Chinese Academy of Sciences, Beijing 100029, China

[4] Department of Land, Air and Water Resources, University of California, Davis, CA, USA

[5] State Key Laboratory for Quality and Safety of Agro-Products, International Science and Technology Cooperation Base for the Regulation of Soil Biological Functions and One Health of Zhejiang Province, Ningbo University, Ningbo 315211, China

[6] State Key Laboratory of Loess and Quaternary Geology, Institute of Earth Environment, Chinese Academy of Sciences, Xi'an, 710061, China

Correspondence: 110079@nnnu.com(M.X.); wangcuriel@foxmail.com(C.W.)

Authors'Email: max_0205@163.com(X.M,); 2213200123@st.gxu.edu.cn(X.O.); chend2022@163.com(D.C.); liyong_L_Y@yeah.net(Y.L.); cmcmillan@ucdavis.edu(C.M.); getida@nbu.edu.cn(T.G.); liuji17@mails.ucas.ac.cn(J.L.); 20200100@gxu.edu.cn(W.S.)

Materials and methods

Soil sampling and analysis

The pH was determined with a pH meter (Metro-pH320; Mettler-Toledo Instruments Shanghai Co. Ltd., Shanghai, China) at a soil: water ratio of 1:2.5 (W: V). Bulk density (BD) was determined gravimetrically by oven-drying fresh soil at 105 °C for 8 hours. NH_4^+-N and NO_3^--N were extracted using 0.5 mol L^{-1} K_2SO_4 and measured with a flow-injection auto-analyzer (Tecator FIA Star 5000 Analyzer, Foss Tecator, Sweden). Soil physicochemical characteristics were determined according to the methods of Bao [1]. Soil microbial biomass carbon (MBC), nitrogen (MBN) were determined by the chloroform fumigation and extraction method, and the conversion factor of 0.45 was used to calculate MBC and MBN contents [2]. The soil MBC contents were measured with a total organic carbon analyzer (TOC-VWP; Shimadzu Corporation, Kyoto, Japan) and MBN were determined by a flow-injection auto-analyzer (Tecator FIA Star 5000 Analyzer, Foss Tecator, Sweden). For SOC analysis (g SOC kg^{-1} dry soil), a sub-sample was ground in a mortar and pestle to pass a 0.149-mm sieve. Organic carbon was determined by dichromate oxidation [3]. TN concentration was estimated using a flow-injection auto-analyzer (Tecator FIA Star 5000 Analyzer, Foss Tecator, Sweden) after being digested by H_2SO_4 and catalysts. Soil TP contents were determined using the molybdenum blue-ascorbic acid method with a spectrophotometer (Lambda 25 UV/VIS spectrophotometer, USA) [4].

References

1. Bao, S.D. Soil and Agricultural Chemistry Analysis. Agriculture Press: Beijing, China, 2005.

2. Wu, J.S.; Jorgensen, R.G.; Pommerening, B.; Chaussod, R.; Brookes, P.C. Measurement of soil microbial biomass-C by fumiga-tion-extraction-an automated procedure. Soil Biol. Biochem. 1990, 22 (8), 1167-1169. doi:10.1016/0038-0717(90)90046-3.
3. Walkley, A.; Black, I.A. An examination of the Degtjareff method for determining soil organic matter, and a proposed modi-fication of the chromic acid titration method. Soil Sci.1934, 37(1), 29-38. doi:10.1097/00010694-193401000-00003.
4. Olsen, S.R.; Cole, C.V.; Watanabe, F.S.; Dean, L.A. Estimation of available phosphorus in soils by extraction with sodium bicarbonate. USDA Circular No. 939. U.S. Government printing Office, Washington, D.C. 1954.

Table S1 The granulometric composition of the soil in different treatments. Mean ± SE.

	F	FT-CK	FT-N	FT-O
Sand (%)	52.47 ± 5.12	66.08 ± 5.92	64.32 ± 5.37	48.8 ± 4.28
Silt (%)	20.9 ± 3.67	15.67 ± 4.10	15.97 ± 4.39	26.7 ± 2.61
Clay (%)	26.63 ± 5.98	18.25 ± 4.55	19.71± 4.64	24.5 ± 5.42

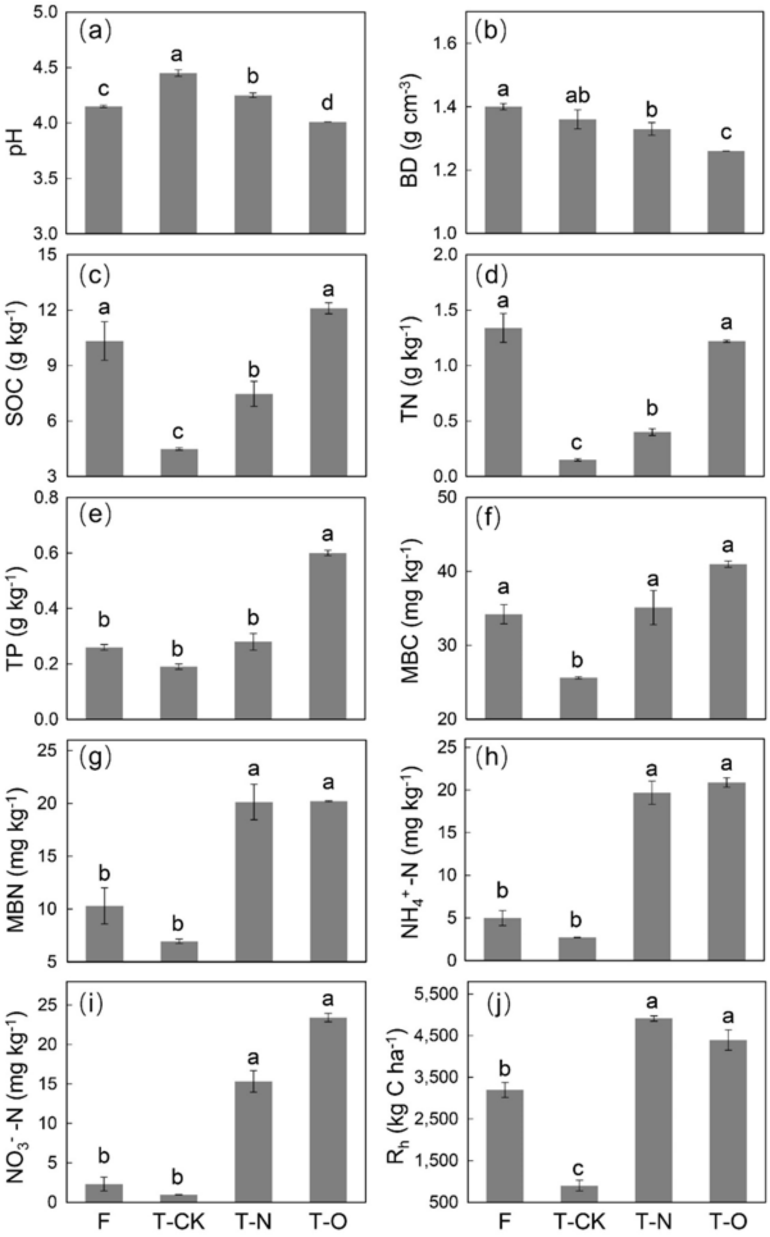

Figure S1 Soil environmental properties. pH, soil bulk density (BD), soil organic carbon (SOC), total nitrogen (TN), total phosphorus (TP), ammonium-N (NH_4^+-N), nitrate-N (NO_3^--N) and soil heterotrophic respiration rate (Rh) of F and FT-N treatments were obtained from Chen et al. (2021). F (Masson pine forests), FT-CK (Forest-to-Tea conversion without fertilization), FT-N (Forest-to-Tea conversion with conventional fertilization), FT-O (Forest-to-Tea conversion for 30 years). Different letters represent significant differences between the treatments ($p < 0.05$). Vertical bars indicate standard errors (n = 3).

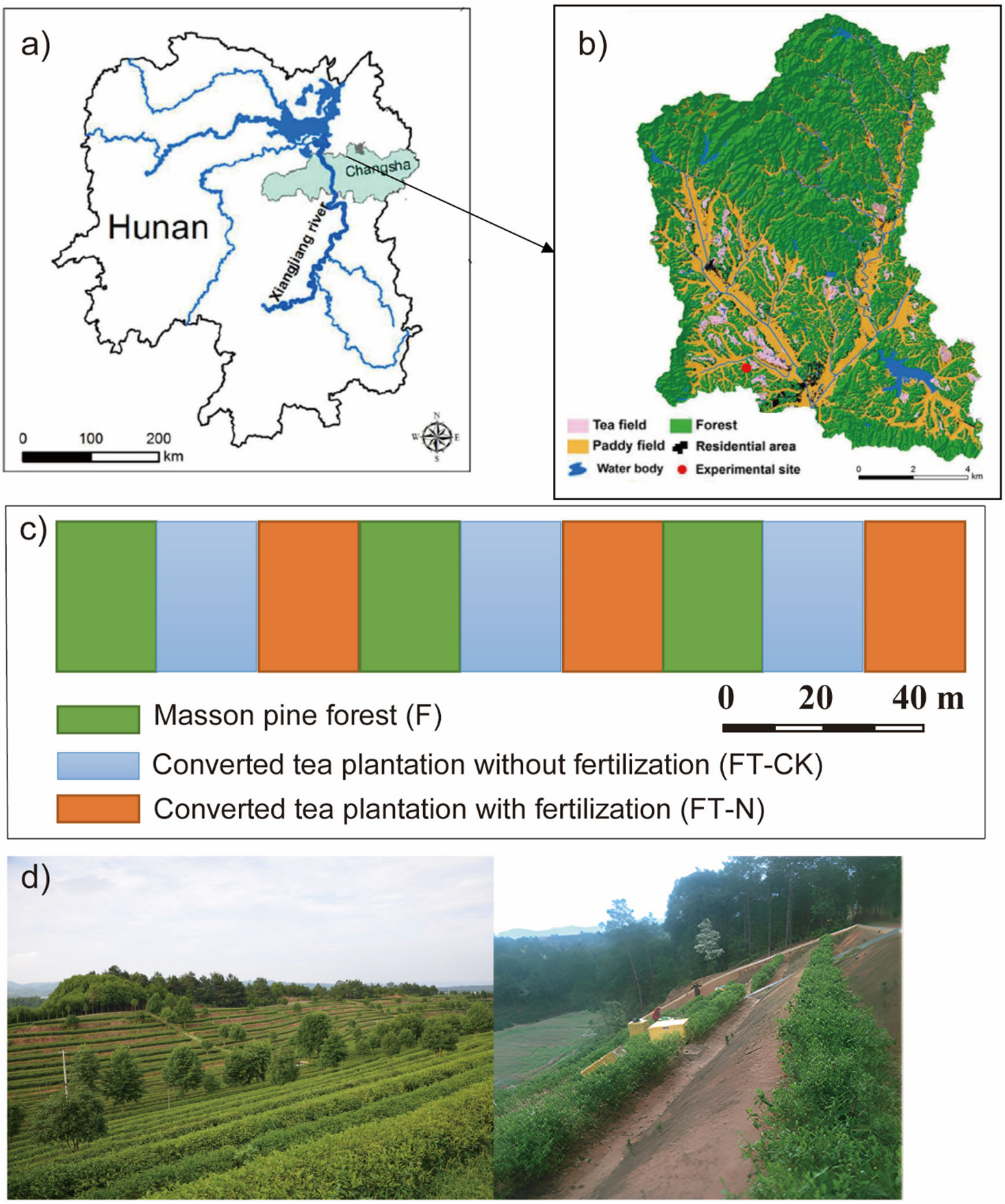

Figure S2 (a) and (b) Geographical location of the study area in Changsha County, Hunan Province, China; (c) Field experimental plots using a randomized block design with three replicates; (d) Photos of the field experiment in study.

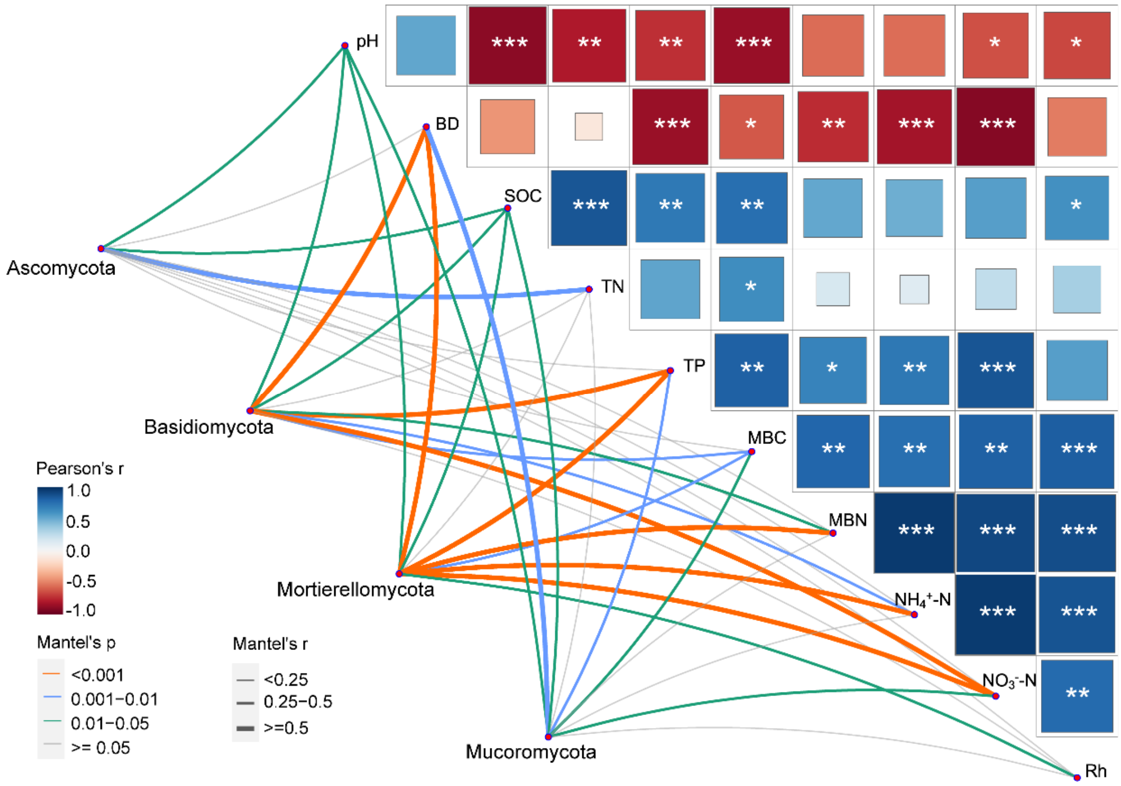

Figure S3 Associations among soil environmental factors and abundant fungi phylum in four treatments. Pairwise comparisons of soil environmental factors and abundant fungal taxa are shown, with a colour gradient denoting Spearman's correlation coefficient. Fungal phylum compositions were related to soil environmental factors by a Mantel test. Edge width corresponds to the Mantel's r statistic for the corresponding distance correlations, and edge color denotes the statistical significance based on 999 permutations.

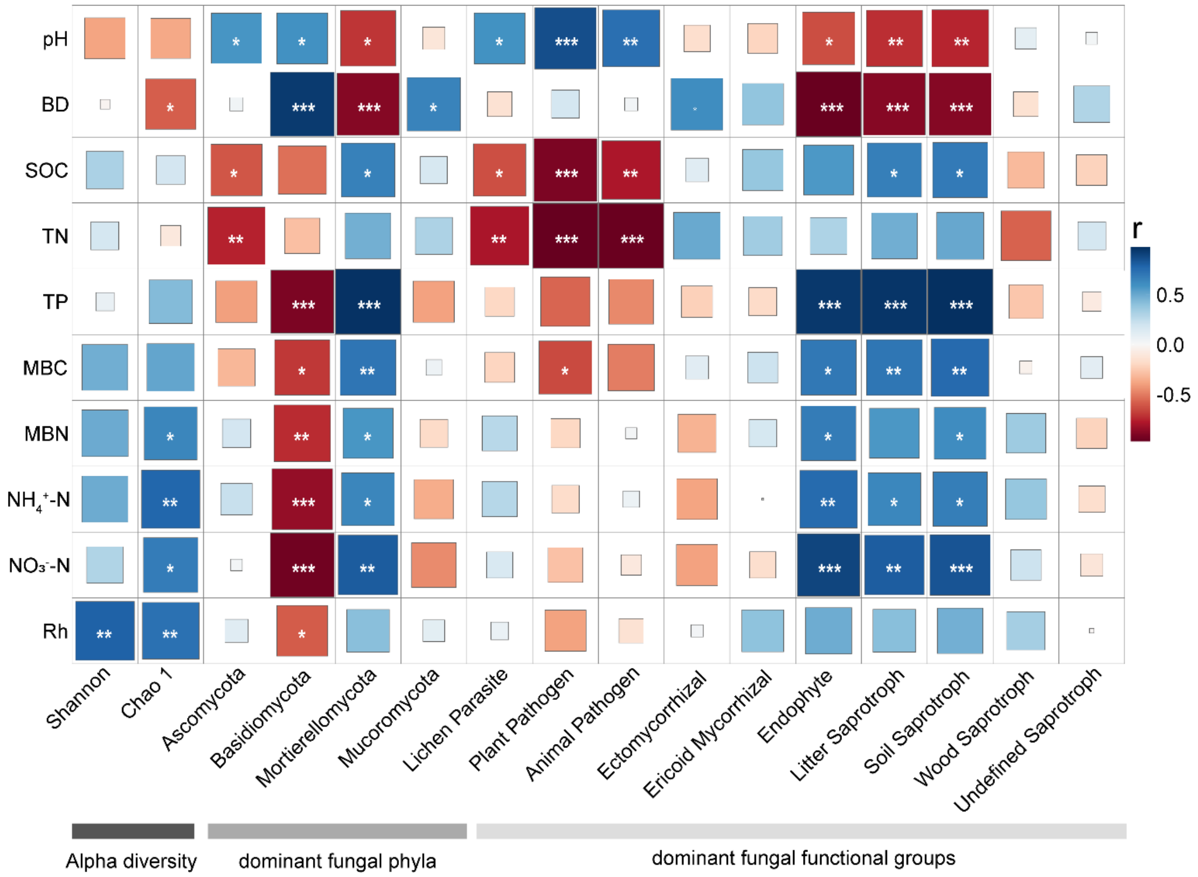

Figure S4. Pearson correlations (r) between soil environment factors and soil fungal alpha diversity (Shannon and Chao 1 index), the relative abundance of dominant fungal phyla and functional groups. BD: Bulk density; SOC: soil organic carbon; TN: total nitrogen; TP: total phosphorus; MBC: soil microbial biomass carbon; MBN: soil microbial biomass nitrogen; NH_4^+-N: ammonium-nitrogen; NO_3^--N: nitrate-nitrogen; Rh: Soil heterotrophic respiration rate.
